# Supplementary material for: Compact, reconfigurable, and scalable photonic neurons by modulation-and-weighting microring resonators
Source: eLight. 2026 Feb 9;6(1):6. doi: 10.1186/s43593-026-00122-3 (PMC12886301; doi:10.1186/s43593-026-00122-3)
Supplement: Supplementary file 1 [file 43593_2026_122_MOESM1_ESM.pdf]

Supplementary Material

Compact, Reconfigurable, and Scalable Photonic Neurons by Modulation-and-Weighting Microring Resonators

Weipeng Zhang[1,2*†], Yuxin Wang[1†], Joshua C. Lederman[1], Bhavin J. Shastri[3] and Paul R. Prucnal[1*]

[1]Department of Electrical and Computer Engineering, Princeton University, Princeton, 08544, New Jersey, USA.*
[2]LightXcelerate, Inc., Palo Alto, 94301, California, USA.
[3]Department of Physics, Engineering Physics and Astronomy, Queen's University, Kingston, K7L 3N6, Ontario, Canada.

[*]Corresponding author(s). E-mail(s): weipeng@lightxcelerate.com; prucnal@princeton.edu;
Contributing authors: yuxinw@princeton.edu; joshuacl@princeton.edu; shastri@ieee.org;
[†]These authors contributed equally to this work.

1. Source of Nonlinearity

The nonlinear activation in our photonic neuron primarily originates from the electro-optic transfer function of the microring resonator (MRR). As also described in the silicon photonic modulator neuron architecture [1], the MRR exhibits a Lorentzian transmission profile with a strongly voltage-dependent resonance shift. When the input voltage perturbation is small, the operating point remains within a locally linear region of this curve. However, for larger voltage excursions, the operating point sweeps across different slopes of the Lorentzian resonance, producing a distinctly nonlinear input-output relationship.

In addition to this primary mechanism, forward-biasing the PN junction introduces free-carrier absorption and self-heating, which further shifts the resonance. This thermal-electrical feedback enhances the curvature of the transfer response and effectively reduces the required electrical swing for achieving nonlinear activation. This same phenomenon is discussed in [1] as a secondary nonlinear mechanism in modulator-class photonic neurons. Together, these effects yield tunable nonlinear transfer functions, including sigmoid-like, ReLU-like, and peaked responses, depending on the relative detuning between the pump wavelength and the MRR resonance. Transfer-curve measurements demonstrating these behaviors are included in the main text and supplementary figures.

In our photonic neuron, the nonlinear activation arises from the electro-optic response of the MRR. This behavior is consistent with previously demonstrated modulator-class photonic neurons, particularly the silicon photonic modulator neuron reported previously [1].

The nonlinear activation observed in our photonic neuron arises primarily from the electro-optic modulation characteristics of the MRR. The transmission spectrum of a microring exhibits a Lorentzian response, and variations in the applied electrical drive shift the resonance wavelength, thereby producing a nonlinear mapping from voltage to optical transmission. This is the exact physical mechanism responsible for nonlinear activation in the silicon photonic modulator neuron demonstrated previously [1], and our device follows an analogous operating principle.

The transmission of the MRR near its resonance wavelength can be modeled by a standard Lorentzian function,

$$T_{MRR}(\lambda) = T_0 \left[1 - \frac{1}{1+\left(\frac{2(\lambda-\lambda_0)}{\Delta\lambda}\right)^2} \right] \tag{S1.1}$$

where T_0 denotes the off-resonant transmission, λ_0 is the resonance wavelength, and $\Delta\lambda$ is the full-width at half-maximum (FWHM). The resonance wavelength is sensitive to the refractive index of the waveguide, and therefore to the voltage applied across the PN junction. To first order, the resonance shift may be approximated as

$$\lambda_0(V) = \lambda_0(0) + \left(\frac{\delta\lambda_0}{\delta V}\right) V \tag{S1.2}$$

reflecting the free-carrier dispersion produced by carrier injection or depletion. Even in the absence of strong carrier accumulation, this shift causes the pump wavelength to move across regions of the Lorentzian with different slopes, and thus the resulting transmission becomes a nonlinear function of the applied voltage. The output optical power may therefore be written as

$$T_{out}(V) = T_{MRR}\left(\lambda_{pump} - \frac{\delta\lambda_0}{\delta V}V\right) \tag{S1.3}$$

which directly illustrates that the nonlinearity originates from the intrinsic curvature of the MRR spectral response.

In addition to this electro-optic mechanism, there is a secondary nonlinear contribution arising from the generation of free carriers and subsequent self-heating under forward-bias operation. When the junction is biased into moderate forward conduction, the circulating optical power inside the ring induces free-carrier absorption, producing a carrier density that contributes to an additional index perturbation. Carrier recombination and absorption also lead to localized heating, which shifts the resonance according to

$$\Delta\lambda_{th} = \left(\frac{dn}{dT}\right)\Delta T \tag{S1.4}$$

where ΔT represents the local temperature rise arising from optical absorption and dn/dT is the thermo-optic coefficient of silicon. Because both free-carrier absorption and thermal diffusion depend on the optical power circulating within the resonator, these processes introduce an additional intensity-dependent resonance shift that reinforces the intrinsic Lorentzian nonlinearity. This effect reduces the electrical voltage swing required to reach the nonlinear regime [1].

Combining the electro-optic and thermal contributions, the overall activation function of the neuron can be expressed as

$$f(V) = T_{MRR}\left(\lambda_{pump} - \Delta\lambda_{EO}(V) - \Delta\lambda_{th}(V)\right) \tag{S1.5}$$

where $\Delta\lambda_{EO}(V)$ denotes the voltage-induced free-carrier dispersion and $\Delta\lambda_{th}(V)$ represents the power-dependent thermo-optic shift. The interplay between these two terms produces a tunable nonlinear activation response whose shape depends on the relative detuning between the pump wavelength and the MRR resonance. When the pump wavelength lies on the steepest portion of the Lorentzian, the activation resembles a sigmoid; for slight detuning, the response resembles a rectified linear unit; and when the pump is aligned near the peak of the resonance, a peaked or quadratic activation is observed.

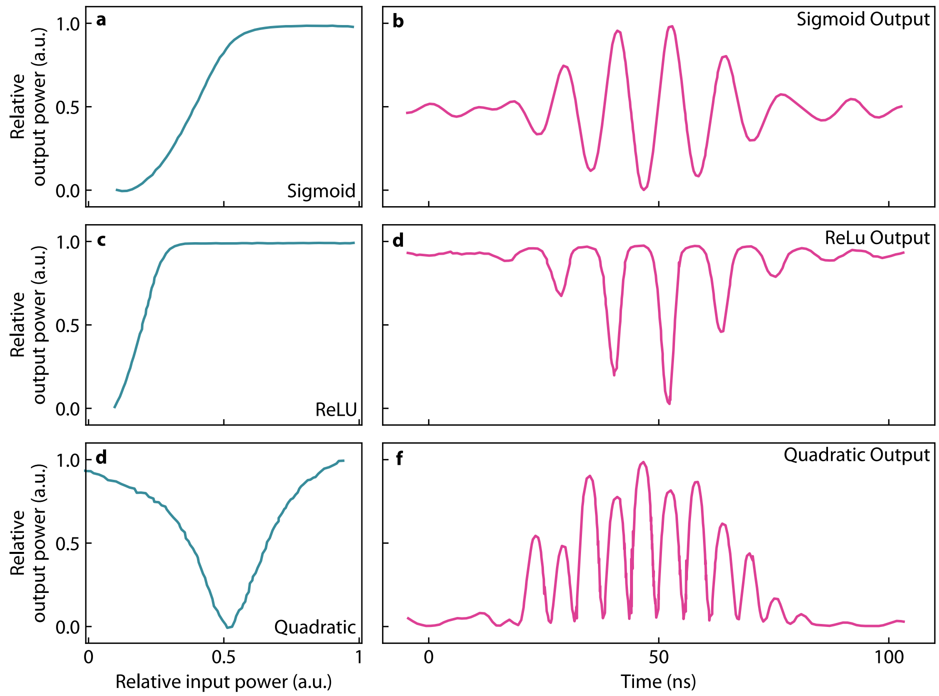

Supplementary Fig. S1. Nonlinear activation by MRR transfer function. The left column is the transfer function, and the right column is the corresponding output. From top to the bottom are nonlinear response for Sigmoid, ReLU and quadratic, respectively, which are by setting different bias point of the MRR.

Supplementary Fig. S1 presents representative measured transfer curves obtained by sweeping the input voltage with a low-frequency sawtooth waveform. The curves illustrate the progression from linear to strongly nonlinear activation as the MRR bias is varied. These measurements confirm that the observed nonlinear activation behavior is a direct consequence of the intrinsic microring transfer function, supplemented by additional feedback from carrier-induced heating under forward bias.

2. Physical Model for Weight Tuning via Thermal and Carrier Effects

The synaptic weight in the proposed modulation-and-weighting architecture is determined by the small-signal modulation efficiency of the MRR at its operating bias point. This efficiency depends on the local slope of the MRR's spectral transmission, which in turn is set by the detuning between the laser wavelength and the ring resonance. To model this quantitatively, we begin with the standard Lorentzian expression for the through-port transmission as in S1.1. The resonance wavelength depends on the tuning currents through the heater and the PN junction. To first order, both mechanisms contribute linearly to the resonance shift, so that

$$\lambda_0(i_{ht}) = \lambda_0^{(0)} + \alpha_{th} i_{ht} \tag{S2.1}$$

where i_{ht} is the heater current, while α_{th} is the thermo-optic tuning efficiency. For a fixed input wavelength λ_L and input power P_{in}, the output power is therefore

$$P_{out}(i_{ht}) = P_{in} T(\lambda_L - \lambda_0(i_{ht})) \tag{S2.2}$$

A small AC modulation $\delta V_{mod}(t)$ applied to the PN junction perturbs the refractive index through the plasma-dispersion effect, inducing a resonance shift. If assume the coefficient $\partial\lambda_0/\partial V_{mod}$ is constant for fixed bias and linearize the tuning relation around the operating point yields

$$\delta P_{out}(t) \approx P_{in} \left.\frac{dT}{d\lambda}\right|_{\text{bias}} \left(\frac{\partial\lambda_0}{\partial V_{mod}}\right) \delta V_{mod}(t) \tag{S2.3}$$

so that the effective optical weight can be written compactly as

$$\omega(i_{ht}) = K \left.\frac{dT}{d\lambda}\right|_{\lambda_L-\lambda_0(i_{ht})} \tag{S2.4}$$

where $K = P_{in}(\partial\lambda_0/\partial V_{mod})$ collects constant prefactors. Eq. S2.4 explicitly shows that the synaptic weight is proportional to the local slope of the Lorentzian, and that the heater and carrier DC biases select the desired slope by adjusting λ_0. This agrees with the measurement shown in Supplementary Fig. S2, where the actual weight shown on the right is proportional to the slope of the transmission shown on the left.

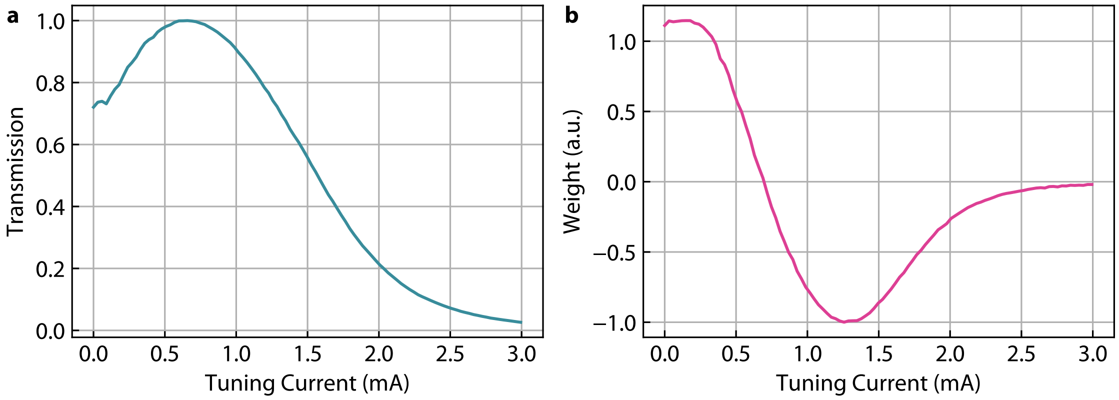

Supplementary Fig. S2. Efficiency of the weight tuning. (a) Transmission of from input to drop port of the test MRR. (b) Measured output weight for the test MRR under the proposed modulation-and-weight usage.

The linearity of the weight with respect to the input modulation follows from the small-signal approximation implicit in Eq. (S2.5). Expanding the Lorentzian to second order yields

$$T(\lambda) \approx T(\lambda_b) + \left.\frac{dT}{d\lambda}\right|_{\lambda_b} \delta\lambda + \frac{1}{2}\left.\frac{d^2T}{d\lambda^2}\right|_{\lambda_b} \delta\lambda^2 \tag{S2.5}$$

The second-order term governs distortion, and its magnitude scales with $\delta\lambda^2$. In the operating regime used here, the modulation voltage swing output from the FPGA is small (about 100mV), and the induced $|\delta\lambda|$ of 0.07 GHz is a tiny fraction of the $\Delta\lambda$ (3.2 GHz). Thus, the quadratic term remains more than an order of magnitude smaller than the linear term. The measured modulation waveforms therefore show negligible distortion, confirming that the synaptic weight is linear in the modulation amplitude under our operating conditions.

The stability of the weight is dominated by the stability of the detuning $\lambda_L - \lambda_0$. The laser wavelength λ_L is well maintained by an internal feedback control loop. Because λ_0 depends directly on temperature via the thermo-optic coefficient of silicon, the chip temperature is regulated using a TEC, which maintains the resonance position with high stability. Any residual slow drift can be removed through occasional recalibration. For applications requiring higher precision, a dithering-based resonance tracking method can be employed. Using this approach, weight precision exceeding 9 bits was previously demonstrated in a two-MRR synapse system [2]. The same mechanism applies directly here because the physical tuning processes are identical.

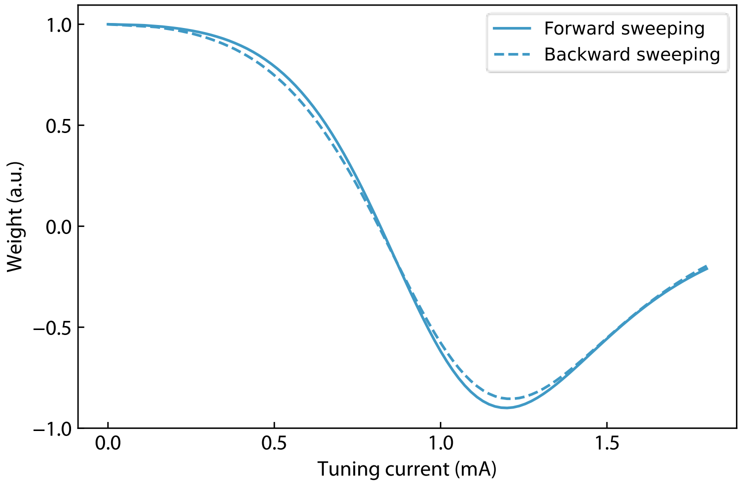

Supplementary Fig. S3. Observation of hysteresis. The actual weight of the MRR during a forward and backward current cycle.

Finally, the operating regime is chosen to avoid thermal bistability or hysteresis, which can occur when the circulating optical power becomes sufficiently large to induce nonlinear self-heating. Under the modest optical powers and voltage swings used here, the thermal feedback remains in the linear regime, and the mapping between tuning currents and weight is single-valued. Forward-reverse bias sweeps presented in Supplementary Fig. S3 show no observable hysteresis, consistent with the model (Eq. S2.4), in which the tuning coefficients are linear, and the device operates away from the bistable regime.

3. Implementation and Calibration of the Recurrent Feedback Delay

In the recurrent configuration used for the financial-prediction experiment, the feedback delay is implemented entirely in the electrical domain after photodetection. The photodetector integrated on the photonic integrated circuit (PIC) produces an electrical photocurrent as its native output, which is routed by wire bonds to a discrete transimpedance amplifier (TIA) mounted on the printed circuit board (PCB). The TIA voltage output is then brought to an SMPM high-frequency connector on the edge of the PCB, and the electrical inputs that drive the MRRs of the modulation-and-weighting bank are likewise delivered through SMPM connectors. This arrangement enables a straightforward construction of the recurrent loop: the output connector of the TIA is physically reconnected to the desired MRR input connector using a coaxial cable whose length determines the feedback delay.

The delay introduced by the coaxial feedback line, is given by

$$\tau_d = \frac{L}{v_p} \tag{S3.1}$$

where L is the physical cable length and v_p is the propagation velocity inside the cable. For the RG-316 coax used in our setup, $v_p \approx 0.66\text{c}$, where c is the speed of light in vacuum. Numerically,

$$L = v_p \tau_d \approx 0.66c \times 1\text{ns} \approx 20 \text{ cm} \tag{S3.2}$$

So a 20 cm coaxial line provides a delay close to 1 ns symbol period at 1 GS/s. The cables were measured and trimmed to this target length. The overall accuracy of the resulting delay is well within the tolerance of the recurrent model, as temporal neural systems trained in situ can compensate for moderate deviations in delay or phase. The recurrent architecture used in this work draws on principles similar to delay-based reservoir computing and recurrent photonic networks, where the precise value of the delay does not need to be exact provided the system is trained with the true hardware response.

Although optical delay elements could in principle be implemented using extended waveguide paths, this approach is incompatible with the present architecture. Because the photodetector directly converts the optical signal into an electrical photocurrent, reinjecting the delayed signal optically would require an optical regeneration stage beyond the scope of the integrated design. More importantly, electrical delay lines enable independent tuning of the feedback delay across different recurrent nodes. For instance, if two MRRs are designated as recurrent elements, the TIA output may be split electrically, and two separate coaxial delay lines of different lengths may be inserted before reaching the respective MRR inputs. Optical delays, by contrast, would add the same physical propagation delay to all MRRs sharing that optical path and therefore could not provide the necessary node-specific configurability.

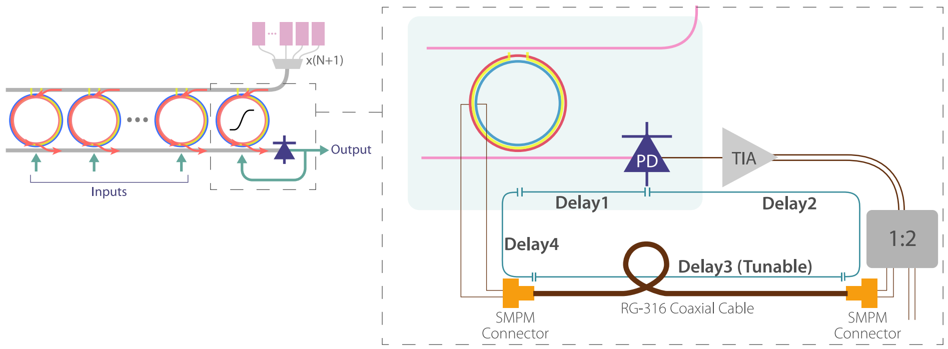

Supplementary Fig. S4. schematic overview of the electrical feedback path

If higher precision in delay alignment is required, the coaxial line may be cut slightly short and an inline RF phase shifter inserted to tune the delay continuously. This hybrid approach combines coarse delay provided by the cable length with fine delay adjustment provided by the phase shifter, enabling sub-sample alignment with the 1 GSPS data stream when necessary. Supplementary Fig. S4 provides a schematic overview of the electrical feedback path, including the integrated photodetector, TIA, SMPM interconnects, and coaxial delay implementation.

4. Inter-Channel Crosstalk in the 10-MRR WDM Bank

To evaluate inter-channel crosstalk in the microring-based WDM bank, we measured the static transmission spectra of all ten MRRs at an operating temperature of approximately 25 °C. A Lorentzian profile well described each resonance, and the FWHM linewidths extracted from these fits were about 3.2 GHz. Supplementary Fig. S5 illustrates the measured resonance centers of the ten MRRs, from which the minimum spacing between adjacent channels was found to be approximately 50 GHz.

The crosstalk between two channels can be estimated directly from the overlap of their Lorentzian lineshapes. If the transmission spectrum of each ring is written as S1.1, with ω denoting the measured FWHM, then the power leakage from channel 2 into the center wavelength of channel 1 is

$$\chi_{1\leftarrow 2} = \frac{T_2(\lambda_1)}{T_2(\lambda_2)} = \frac{1}{1+\left(\frac{2(\lambda_1-\lambda_2)}{\omega_2}\right)^2} \tag{S4.1}$$

Using the measured values of ω_2 and $|\lambda_1 - \lambda_2|$, the resulting inter-channel leakage evaluates to approximately 0.1 % of the peak transmission of the neighboring channel. Expressed in logarithmic units, this corresponds to a worst-case crosstalk level on the order of −30 dB. Supplementary Fig. S5 shows the measured resonance pair with the corresponding Lorentzian fits, as well as the modeled overlap that yields the crosstalk value quoted here.

No measurable degradation in the system's performance was observed because of this residual leakage. This is consistent with the fact that the linewidth-to-spacing ratio $\omega/\Delta\lambda_{\text{spacing}}$ is significantly below unity (typically $\sim 10^{-2}$), ensuring passive spectral isolation between adjacent channels. Under these conditions, additional crosstalk-mitigation mechanisms—such as active wavelength locking, guard-banding, or digital equalization—are unnecessary. Nevertheless, if future applications demand even denser channel packing or higher dynamic range, any of the standard stabilization techniques used in high-order MRR-WDM systems can be incorporated without modifying the fundamental architecture presented in this work.

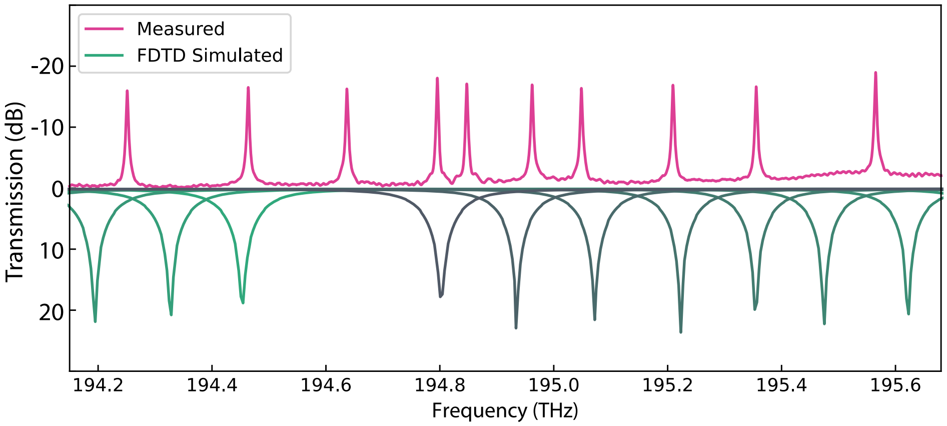

Supplementary Fig. S5. Measured spectra and FDTD simulated spectra of the 10 MRRs.

To further validate the spectral fidelity of the MRRs used in the convolution kernel, we compared the measured transmission spectra of the rings with the FDTD-simulated profiles, as shown in the lower half of Supplementary Fig. S5. The model closely reproduces the expected spectral shapes, with noticeable deviations attributable to standard fabrication tolerances. Since each MRR is operated at a wavelength set by the measured resonance (via per-channel laser tuning), these minor deviations do not affect the implemented convolution kernel.

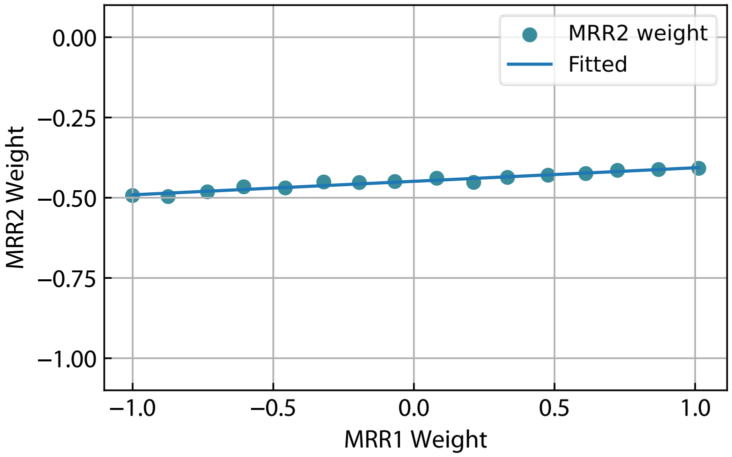

Supplementary Fig. S6. Measured crosstalk between MRRs.

In addition to the spectral-overlap analysis presented above, we experimentally quantified thermal crosstalk by sweeping the heater of a selected MRR while monitoring the resonance of its nearest neighbor. As shown in Supplementary Fig. S6, driving one ring across its full tuning range produced a weight shift of about 5% in the adjacent ring. This confirms that the thermal interaction between neighboring MRRs at sub-mW heater power is negligible and does not contribute meaningfully to inter-channel crosstalk. Together with the -30 dB spectral leakage extracted from the Lorentzian-overlap model, these measurements demonstrate that both thermal and spectral isolation are sufficient for the 10-element array and support straightforward scaling to larger banks with similar device parameters.

5. Full Power Consumption Breakdown

The average per-element power consumption quoted in the main text (0.186 mW) refers exclusively to the on-chip tuning and modulation power of the photonic integrated circuit. This value includes the steady-state heater power required to position each MRR at its designated operating wavelength, as well as the DC current applied to the PN junction to set the electro-optic modulation bias. No electronic instrumentation or off-chip driver power is included in this number, as those contributions depend strongly on implementation choices and are not intrinsic to the photonic architecture itself.

For completeness and transparency, we provide here an analysis of the power consumed by the system during the experiments. Supplementary Table S1 summarizes the measured and estimated contributions from each subsystem. The “tuning power” includes the heater and PN-junction power, summed across all 10 MRRs. The TIA and RF-driver figures represent the quiescent and

dynamic power of the discrete components used in our laboratory setup. The FPGA values include both the total board power reported by the device telemetry and the estimated fraction attributable to the high-speed I/O banks that directly drive the MRRs. Because the FPGA runs a complete Linux environment, soft peripherals, and background processes unrelated to photonic computation, the majority of its power draw does not meaningfully contribute to the photonic neural network operation. Only the I/O switching power is relevant to link-level operation, and this is listed separately in the table.

The numbers indicate that the intrinsic on-chip contribution—the heater and PN-junction power—is two to three orders of magnitude smaller than the external electronic overhead. This distinction is essential for interpreting energy efficiency: the power for the PIC is scalable and fundamental to the photonic synaptic elements, whereas the electronic overhead is essentially implementation specific and can be reduced substantially through tighter integration, ASIC- or driver-level optimization, or co-packaged electronics. The values reported here, therefore, represent a laboratory measurement setup rather than a lower bound on system-integrated power.

Supplementary Table S1. Total power consumption breakdown of the experimental setup.

	Component / Subsystem	Power Consumption (per MRR)	Power Consumption (total)	Notes
**PIC**	MRR, heaters	0.186 mW	1.86 mW	330 Ohms, 0-1.3 mA tuning range, average power
	MRR, PN-junction bias	0.055 µW	0.55 µW	$I_{DC} = 50$ nA, $V_{\text{bias}} = -1.1\text{V}$
	MRR, PN-junction modulation	1.06 µW	10.6 µW	100 mV modulation voltage swing, $C_j = 40$ fF
	PD, bias	-	0.11 µW	$I_{dark} = 100$ nA, $V_{\text{bias}} = -1.1\text{V}$
**FPGA**	RF DAC	-	1.0 W	AMD Inc, ZU49DR, Vivado software estimation
	RF ADC	-	0.4 W	Same as above
	Processor	-	1.2 W	Same as above
**Misc**	Thermal tuning DAC	-	26.5 mW	Analog Device Inc, LTC2662
	TIA	-	1.21 W	Analog Device Inc, HMC7590

6. Quantitative Comparison with Prior Integrated Photonic Neural Processors

To contextualize the performance of the demonstrated MRR-based photonic neuron and modulation-and-weighting bank, we compiled key figures of merit reported in prominent integrated photonic neural and neuromorphic computing demonstrations. Supplementary Table S2 summarizes the PIC footprint, experimentally demonstrated computing size, compute speed, compute density, and energy efficiency. For consistency, each row adopts the standard definition of compute speed: 2 × Matrix Size × Baud Rate. The table caption details the assumptions used in these calculations. Notably, we include only the costs of major photonic components, including modulators, weighting elements, and photodetectors (PD). We exclude electronics digital-to-analog converters (DAC), analog-to-digital converters (ADC) and laser power, which vary substantially across vendors.

This comparison shows that our architecture delivers competitive compute speed and experimentally validated compute scale, while offering distinctly superior compute density owing to the compact MRR footprint and the integration of modulation and weighting in a single device. Furthermore, the steep tuning curves of our MRRs result in extremely low tuning energy, leading to outstanding overall energy efficiency.

Supplementary Table S2. Comparison of representative photonic neural processors.

Work/ Year	Architecture/ Method	Footprint[a] (mm^2)	Matrix Size	Compute Speed (TOPs)	Compute Density (TOP/s/mm²)	Energy Efficiency[b] (TOPs/W)
**This work**	CNN, RNN/ MRR modulate-n-weight bank	0.042	1x10	0.196 (9.83Gbaud)	4.67	105 (0.196TOPs/1.9mW)
**[3]/ 2017**	General MVM/ MZI mesh	0.67	1x4	40e-9 (10kbaud[c])	59e-9	Low[d]
**[4]/ 2021**	CNN by dispersive delay/ Comb + EOM + long fiber	Large[e]	9x10	11 (65Gbaud)	Low[e]	5.8 (0.477TOPs/82mW)
**[5]/ 2023**	General MVM/ MZI mesh	0.76	4x4	0.032 (1Gbaud)	0.021	0.04 (0.032TOPs/800mW)
**[6]/ 2021**	CNN/ Comb + VOA + Crossbar	1.54+ off-chip VOA	4x9	72e-9 (1kbaud)	46e-9 (on-chip only) Low (w/VOA)	Low[d]
**[7]/ 2023**	Time-multiplexing MVM/ MZM + MRR weight bank	0.832	2x2	0.136 (17Gbaud)	0.163	0.571 (0.136TOPs/238mW)
**[8]/ 2023**	CNN/ MZM (off-chip) + MMI	0.11+ off-chip MZM	3x4	0.398 (16.6Gbaud)	1.8 (on-chip only) Low (w/MZM)	3.98 (0.398TOPs/100mW)
**[9]/ 2025**	Time-multiplexing MVM/ EOM (off-chip) + MZI mesh	29.6+ off-chip MZM	4x4	1.28 (40Gbaud)	0.043 Low (w/QOM)	12.8 (1.28TOPs/100mW)
**[10]/ 2024**	General MVM/ TFLN MZI mesh	0.96	4x4	0.64 (20Gbaud)	0.66	32 (0.64TOPs/21mW)
**[11]/ 2025**	CNN by dispersive delay/ EO comb + MZM + FBG delay	6.7	3x9	1.62 (30Gbaud)	0.24	324 (1.62TOPs/5mW)

a. All photonic components (modulator, weighting element, PD, etc), exclude laser source, electrical routing, and optical couplers. If exact numbers not explicitly given, the size is measured using the chip micrograph and the scale bar.
b. Photonic components only (modulator, weighting element, PD, etc), exclude laser power
c. Assumed as typical performance for thermal tuned phase shifter.
d. The modulation speed is low
e. This is not an integrated setup with many off-chip discreet components.

7. Modulation Bandwidth of the MRR Modulators

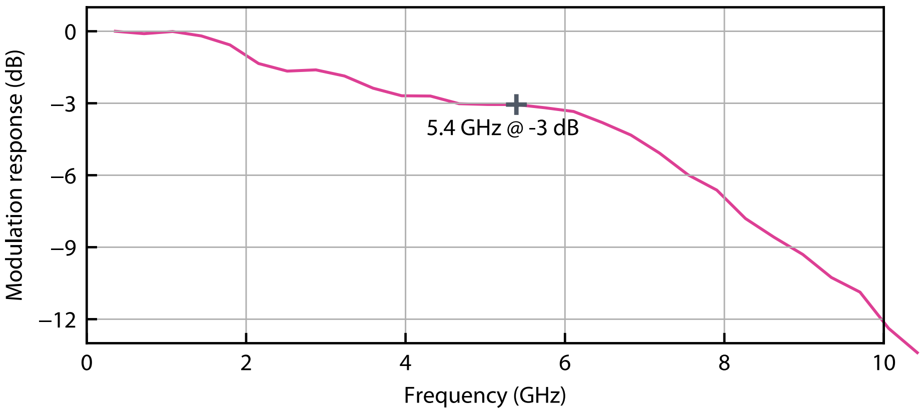

Supplementary Fig. S7. Modulation bandwidth of MRR. The 3dB bandwidth is 5.4 GHz.

The electro-optic modulation bandwidth of the MRR's PN-junction modulator was measured using a small-signal S21 frequency sweep. A swept RF excitation was applied to the modulator, and the optical output was detected by a high-speed photodiode and analyzed with a vector network analyzer. Supplementary Fig. S7 shows the measured EO response for one representative modulator. The extracted 3-dB bandwidth is 5.4 GHz. These values sufficiently exceed the 1 GSPS

operation used in the main experiments, confirming that the modulator dynamics do not limit the system performance.

8. Latency breakdown and analysis for HFT application

The latency of the currently implemented system and that of a projected all-analog photonic system are summarized in Supplementary Table S3. As illustrated in Fig. 4(a), a typical trading workflow begins with requesting market data from an exchange, which is currently provided in digital form. In addition to data-transmission latency over Ethernet, the total processing latency can be decomposed into three components: (1) electronic processing of the digital input data, (2) low-latency computation on the PIC, and (3) electronic processing (thresholding) of the output data before transmission back to the exchange. Because the input and output data are digital, while the computation within the PIC is performed using analog optical signals, electronic-to-optical (E/O) and optical-to-electronic (O/E) conversions, implemented with MRRs and PDs, are required and introduce additional latency.

For digital-to-analog and analog-to-digital conversion, the intrinsic conversion latency of the current-steering DAC and the 16-bit pipeline ADC is relatively small. Instead, the dominant contribution arises from the AXI-stream fabric within the FPGA, which dispatches and pipelines data to the DACs and ADCs. In our setup, the AXI-stream fabric operates at 491.5 MHz. It typically requires approximately five clock cycles for the DAC path and ten clock cycles for the ADC path, corresponding to latencies of about 10 ns and 20 ns, respectively.

Quantifying the digital processing latency analytically is nontrivial, as it depends on operating-system scheduling, Python execution overhead, and memory-access traffic on the ARM processor. We therefore measure the aggregate processing time empirically by directly timing program execution. To mitigate run-to-run variability arising from background processor load, the same program is executed ten times, and the average latency is reported. Under these conditions, the measured digital processing latency is approximately 300 ms.

A more effective and industry-standard approach is to implement the required thresholding and decision logic directly in the FPGA fabric rather than on the ARM processor using Python. In such a hardware implementation, the computation can be performed deterministically in a single clock cycle. Although this optimization is not implemented in the present setup, the corresponding latency can be readily estimated as one fabric clock period, which is approximately 2 ns for a 500 MHz clock.

The photonic processing latency comprises contributions from E/O and O/E conversion and optical propagation within the PIC. While conversion latencies are broadly comparable across implementations, the propagation delay depends on the PIC layout and the physical separation between modulators and photodetectors. In our design, this distance is minimized: modulation and weighting are performed within the same MRR, and the photodetector is placed immediately after the MRR bank. The maximum optical path length, which is from the first MRR to the PD, is approximately 2.1 mm, corresponding to a propagation delay of about 26 ps assuming a group refractive index of 3.8.

The modulation latency of the MRR can be estimated as

$$\tau_{\mathrm{MRR}} \approx \frac{Q}{\pi f_0} \approx 98 \text{ ps}$$

where $f_0 = 193.5\text{THz}$ and the measured quality factor is $Q \approx 6 \times 10^4$. The photodetector latency is estimated as

$$\tau_{\mathrm{PD}} \approx \frac{0.35}{\mathrm{BW}} \approx 17 \text{ ps}$$

for a PD bandwidth of 20 GHz. Similarly, the TIA, with a bandwidth of 33 GHz, contributes an estimated latency of

$$\tau_{\mathrm{TIA}} \approx \frac{0.4}{\mathrm{BW}} \approx 12 \text{ ps}$$

Together, the PD and TIA introduce a combined latency of approximately 29 ps.

Supplementary Table S3. Latency analysis

	Item		Current system<br>Photonics-Electronics hybrid	Projected system<br>All analog photonics
**Electronics (Data-in)**	DAC pipeline delays		10 ns	-
**Photonics (Processing)**	Microring modulation		98 ps	98 ps
	On-chip optical propagation		26 ps	26 ps
	Photodetector and TIA response		29 ps	29 ps
	Photonic thresholding		-	105 ps
**Electronics (Data-out)**	ADC pipeline delays		20 ns	-
	Electronic Thresholding	By ARM processor	300 ms	-
		By FPGA logic	2 ns	-
		total	300.032 ms (ARM)<br>32 ns (FPGA)	0.258 ns

For the ultimate low-latency performance, even the thresholding calculation by the FPGA can be improved by implementing it in analog photonics. One viable architecture is a photonic thresholder based on two Mach-Zehnder interferometers and an MRR, as demonstrated in previous work [12]. The latency of this photonic thresholder is the light propagation time plus the additional delay in the MRR, which are 7 ps and 98 ps, respectively, for a total of 105 ps.

From the analyzed latency budget, the dominant contribution in the current implementation arises from electronic processing rather than from the photonic computation itself, as electronic

subsystems are fundamentally limited by clocked or sampled operation. Given that present-day market data requests and trading commands are exchanged in digital electronic formats, A/D and D/A conversion stages are unavoidable in the current system and constitute a major source of latency.

Looking forward, one may consider an alternative architectural scenario in which data interfaces are co-designed with analog or hybrid analog–photonic processing hardware. Under such assumptions, market data could be provided as analog electrical signals, applied directly or with minimal signal conditioning, to the microring modulators. Similarly, the photonic neural network output, obtained as an analog voltage from the photodetector and transimpedance amplifier, could be transmitted without intermediate digitization. In this case, the latency budget would be dominated by photonic processes, including modulation, detection, and optical propagation within the PIC.

Under this projected all-analog operating model, the total end-to-end latency is estimated to be approximately 0.26 ns, as summarized in Supplementary Table S3. While such an interface is beyond the scope of the present experimental demonstration, this analysis provides a quantitative lower bound on the achievable latency of the proposed photonic neuron architecture. It highlights its potential advantage in applications where ultra-low-latency processing is paramount.

9. MRR stability and Effectiveness of in-situ training

MRRs are inherently sensitive to environmental drift, most notably temperature variations, which shift their resonance wavelengths and corresponding tuning curves. If the tuning currents are held fixed, such shifts can degrade the performance of the photonic neural network. While periodic recalibration of the tuning curves can mitigate this effect, in-situ training provides a more effective and performance-oriented approach by directly adapting the MRR weights to the current device conditions. The proposed modulation-and-weight MRR bank is fully compatible with in-situ training, as demonstrated below.

Using the high-frequency trading (HFT) task as an illustrative example, we first set the global chip temperature to 24 °C and trained all ten MRRs to maximize performance. The resulting tuning curves, trained tuning currents, and estimated weights are shown in Supplementary Fig. S8(b) and in the inset of Fig. S8(d). We then increased the controlled chip temperature to 24.5 °C, which caused systematic resonance shifts and corresponding changes in the tuning curves across all MRRs, as shown in Supplementary Fig. S8(a) and (c). When the original tuning currents were retained, the achieved gain for the same input market data decreased due to the drift in the effective weights, as indicated by the green and gray bars in Fig. S8(d).

Subsequently, in situ training was performed by fine-tuning the tuning currents around their previously optimized values. This process converged on a new set of tuning currents that partially compensated for the drift in transfer characteristics, resulting in a recovery of system performance, as shown by the coral-colored bars and curves in Fig. S8(d). These results demonstrate that the initially trained weights provide a good starting point for adaptation and that in-situ training is an effective mechanism for compensating environmental drift in practical operation.

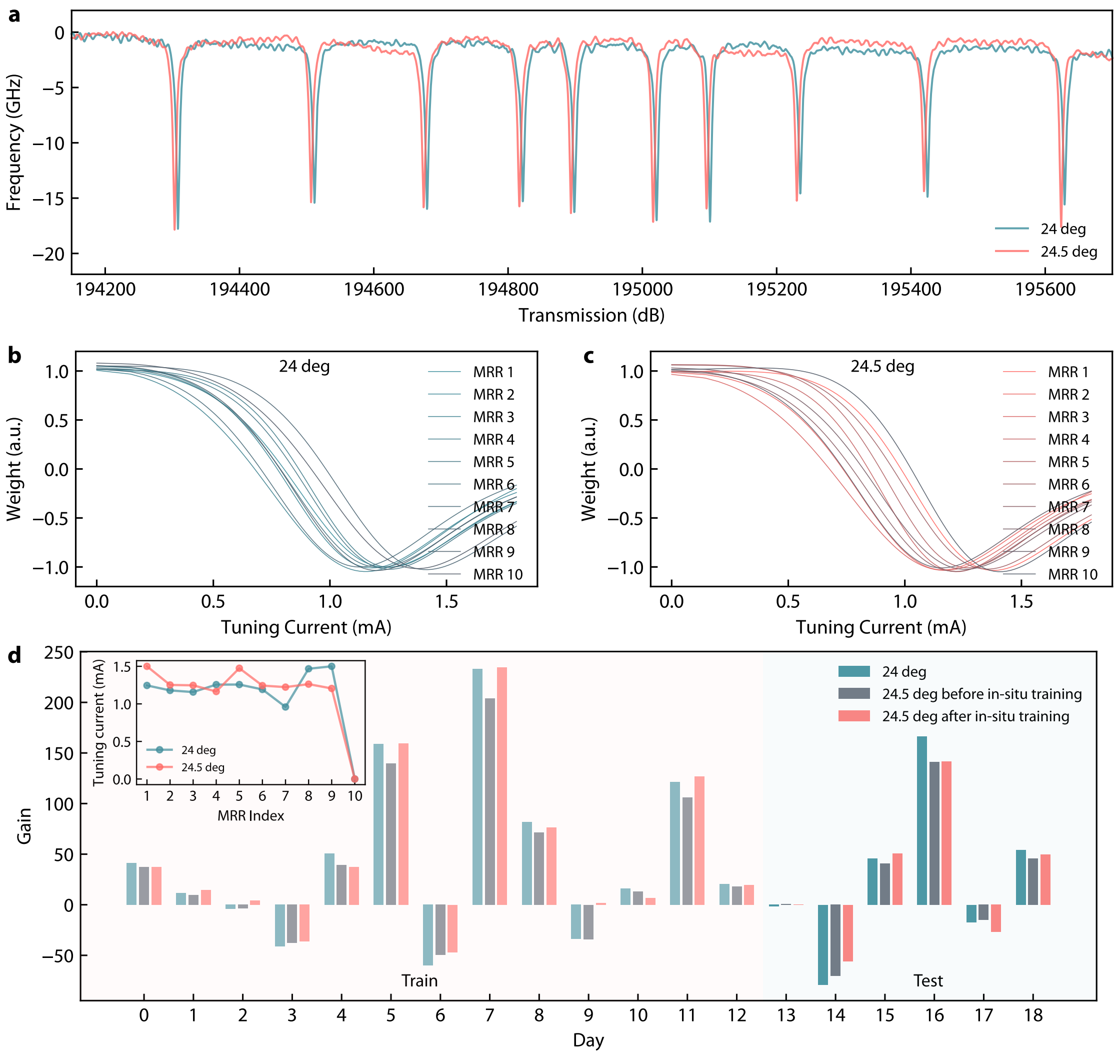

Supplementary Fig. S8. MRR temperature sensitivity and effectiveness of in-situ training. (a) MRR spectra when at 24 degrees and 24.5 degrees PIC temperature. (b) and (c), the tuning curve of all the MRR at 24 deg and 24.5 deg, respectively. (d) The training results for processing recent 18 consequent trading days for stock "GOOG". If keeping the tuning current unchanged, the gain decreased when temperature shift from 24 deg (green bars) to 24.5 deg (gray bars). After in-situ training, the gain recovered with a new set of tuning current applied (coral pink bars).

References

[1] A. N. Tait et al., Silicon photonic modulator neuron. Phys. Rev. Appl. **11**, 064043 (2019).

[2] W. Zhang et al., Silicon microring synapses enable photonic deep learning beyond 9-bit precision. Optica **9**, 579–584 (2022).

[3] Y. Shen et al., Deep learning with coherent nanophotonic circuits. Nat. Photon. **11**, 441–446 (2017).

[4] X. Xu et al., 11 TOPS photonic convolutional accelerator for optical neural networks. Nature **589**, 44–51 (2021).

[5] S. Pai et al., Experimentally realized in situ backpropagation for deep learning in photonic neural networks. Science **380**, 398–404 (2023).

[6] J. Feldmann et al., Parallel convolutional processing using an integrated photonic tensor core. Nature **589**, 52–58 (2021).

[7] B. Bai et al., Microcomb-based integrated photonic processing unit. Nat. Commun. **14**, 66 (2023).

[8] X. Meng et al., Compact optical convolution processing unit based on multimode interference. Nat. Commun. **14**, 3000 (2023).

[9] Y. Xie, X. Ke, S. Hong, Y. Sun, L. Song, H. Li, P. Wang, and D. Dai, Complex-valued matrix-vector multiplication using a scalable coherent photonic processor. Sci. Adv. **11**, eads7475 (2025).

[10] Y. Zheng et al., Photonic neural network fabricated on thin-film lithium niobate for high-fidelity and power-efficient matrix computation. Laser Photon. Rev. **18**, 2400565 (2024).

[11] J. He et al., Programmable electro-optic frequency comb empowers integrated parallel convolution processing, Preprint at https://arxiv.org/abs/2506.18310 (2025).

[12] C. Huang et al., Programmable silicon photonic optical thresholder. IEEE Photonics Technol. Lett. **31**, 1834–1837 (2019).
